# Exploring mixed microbial community functioning: recent advances in metaproteomics

**DOI:** 10.1111/j.1574-6941.2011.01284.x

**Published:** 2012-01-16

**Authors:** Alma Siggins, Eoin Gunnigle, Florence Abram

**Affiliations:** 1 Microbial Ecology Laboratory, Department of Microbiology and Ryan Institute, National University of IrelandGalway (NUI, Galway), Galway, Ireland; 2 Functional Environmental Microbiology, Department of Microbiology, National University of IrelandGalway (NUI, Galway), Galway, Ireland

**Keywords:** environmental proteomics, human gut microbiota, marine and freshwater environment, soil, bioengineered systems

## Abstract

System approaches to elucidate ecosystem functioning constitute an emerging area of research within microbial ecology. Such approaches aim at investigating all levels of biological information (DNA, RNA, proteins and metabolites) to capture the functional interactions occurring in a given ecosystem and track down characteristics that could not be accessed by the study of isolated components. In this context, the study of the proteins collectively expressed by all the microorganisms present within an ecosystem (metaproteomics) is not only crucial but can also provide insights into microbial functionality. Overall, the success of metaproteomics is closely linked to metagenomics, and with the exponential increase in the availability of metagenome sequences, this field of research is starting to experience generation of an overwhelming amount of data, which requires systematic analysis. Metaproteomics has been employed in very diverse environments, and this review discusses the recent advances achieved in the context of human biology, soil, marine and freshwater environments as well as natural and bioengineered systems.

## Introduction

**Fig 1:**
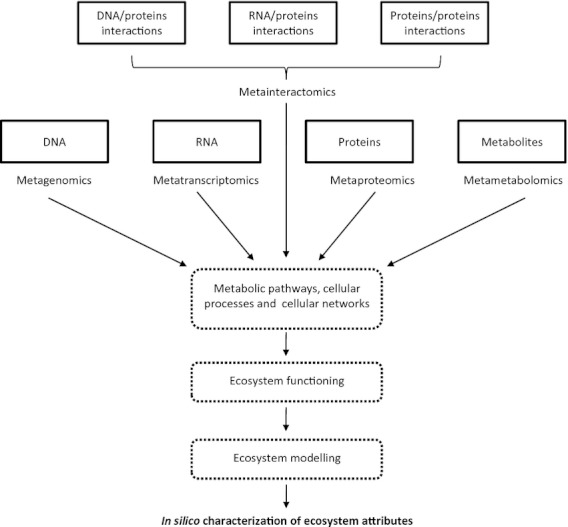
System approach for the characterization of microbial ecosystems. Metagenomics (DNA sequencing of all the microorganisms from an ecosystem), metatranscriptomics (analysis of RNA collectively transcribed by all the microorganisms from an ecosystem), metaproteomics (analysis of proteins collectively expressed by all the microorganisms from an ecosystem) and metametabolomics (analysis of metabolites collectively produced by all the microorganisms from an ecosystem) are employed to access the metabolic pathways and cellular processes at work in an ecosystem. Metainteractomics (analysis of the molecular interactions between all the microorganisms from an ecosystem) is used to investigate the cellular network in an ecosystem. All the resulting data provide insights into ecosystem functioning and are used to generate a model, which in turn can allow the prediction of the behaviour of an ecosystem in response to environmental changes.

Microorganisms occupy virtually every habitat on our planet, and their activities largely determine the environmental conditions of today's world. Indeed, microorganisms are heavily involved in biogeochemistry, ensuring the recycling of elements such as carbon and nitrogen (Madsen, 2011). In addition, microorganisms are extensively used to degrade anthropogenic waste prior to release into the environment (Hussain *et al*., 2010; Park *et al*., 2008b). In their natural habitat, microorganisms coexist in mixed communities, the complexity of which is specific to each environment, for example from six estimated individual taxa for an acid mine drainage biofilm (Ram *et al*., 2005), up to 10^6^ estimated taxa per gram of soil (Wilmes & Bond, 2006). As most of the microorganisms present in the environment have not been cultured, their investigation requires the use of molecular techniques that bypass the traditional isolation and cultivation of individual species (Amann *et al*., 1995). Moreover, even when isolation is possible, a single species removed from its natural environment might not necessarily display the same characteristics under laboratory conditions as it does within its ecological niche. Therefore, the study of mixed microbial communities within their natural environment is key to the investigation of the diverse roles played by microorganisms, and to the identification of the microbial potential for biotechnological application, including but not limited to: pharmaceutical, diagnostics, waste treatment, bioremediation and renewable energy generation. An emerging field of research in microbial ecology encompasses system approaches (Fig. 1), whereby all levels of biological information are investigated (DNA, RNA, proteins and metabolites) to capture the functional interactions occurring in a given ecosystem and identify characteristics that could not be accessed by the study of isolated components (Raes & Bork, 2008; Röling *et al*., 2010). Recent technological advances, including the development of high-throughput ‘omics’ methods, make such system approaches possible, where mixed microbial communities are viewed as one meta-organism. Metagenomics, metatranscriptomics, metaproteomics and metametabolomics are employed to determine respectively the DNA sequences of the meta-organism under study, the collectively transcribed RNA, the translated proteins and the metabolites resulting from cellular processes. All of the generated data can then be used to identify the metabolic pathways and cellular processes at work within an ecosystem. Yet another level of information is required to access the molecular interactions occurring within the ecological niche under investigation, and this is achieved by the application of metainteractomics (Fig. 1; Lievens *et al*., 2010; Medina & Sachs, 2010; Janga *et al*., 2011). Ultimately, system approaches aim to develop mathematical models that can be used to predict the behaviour of a biological system in response to environmental stimuli (Fig. 1; Raes & Bork, 2008; Röling *et al*., 2010).

**Fig 2:**
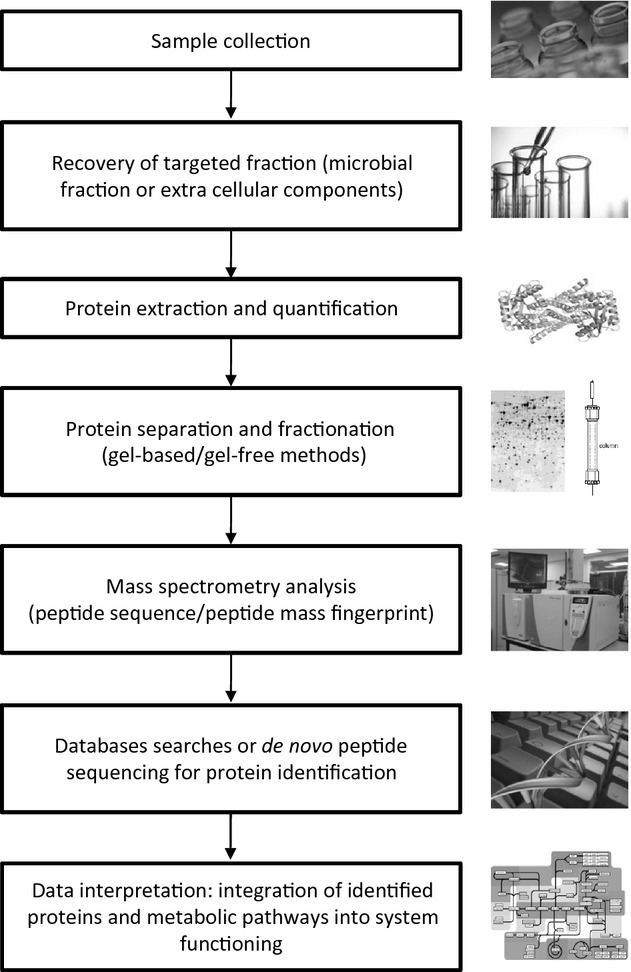
Typical workflow for metaproteomics analysis.

Metaproteomics, which is the identification of all the proteins expressed at a given time within an ecosystem (as defined by Wilmes & Bond, 2004), is an indispensable element of system approaches and plays a key role in the determination of microbial functionality. Microbial metaproteomics has been applied in the context of diverse environments such as soil (Benndorf *et al*., 2007; Williams *et al*., 2010; Wang *et al*., 2011), sediments (Benndorf *et al*., 2009; Bruneel *et al*., 2011), marine (Morris *et al*., 2010; Sowell *et al*., 2011), freshwater (Ng *et al*., 2010; Habicht *et al*., 2011; Lauro *et al*., 2011), human intestinal tract (Verberkmoes *et al*., 2009a; Rooijers *et al*., 2011), human oral cavity (Rudney *et al*., 2010), animal guts (Toyoda *et al*., 2009; Burnum *et al*., 2011) and natural and bioengineered systems (Ram *et al*., 2005; Wilmes *et al*., 2008a; Jehmlich *et al*., 2010). Typically, metaproteomic approaches involve up to seven main steps (Fig. 2), namely sample collection, recovery of the targeted fraction, protein extraction, protein separation and/or fractionation, mass spectrometry analysis, databases searches and finally data interpretation, whereby the expressed proteins and pathways identified are used to access information about system functioning (for detailed descriptions of the methodologies involved, see Wilmes & Bond, 2006 and Verberkmoes *et al*., 2009b). Each environment offers specific challenges and limitations within this workflow. Typically, sample collection and recovery of the targeted fraction (Fig. 2) can be problematic in the marine and freshwater context, where microorganisms can be recovered from hundreds of litres of water away from laboratory facilities. The protein extraction step (Fig. 2) has proven specifically difficult when dealing with soil samples, which naturally contain interfering humic acids. Such compounds are usually co-extracted together with proteins and are known to interfere with protein quantification, separation and identification (Bastida *et al*., 2009). The use of gel-based methods for protein separation presents some disadvantages regardless of the origin of the sample. Such drawbacks are typically those associated with two-dimensional gel electrophoresis (2-DGE): proteins with extreme isoelectric points (basic or acidic) or extreme molecular weight (very large or very small), lipophilic proteins and low abundance proteins are typically excluded (Gygi *et al*., 2000; Ong & Pandey, 2001). Finally, the limitations encountered in the last three steps of the metaproteomic workflow (Fig. 2), namely mass spectrometry analysis, databases searches and data interpretation are intrinsically linked to the success of the previous steps with a disadvantage in fields where no metagenome sequences are available. Overall, metaproteomics relies on the availability of relevant genome and metagenome sequences when searching generated mass spectra against existing databases for protein identification. As such, this approach cannot be viewed as an isolated method because it benefits from genome/metagenome sequencing for protein identification. However, when no relevant sequences are available, *de novo* peptide sequencing can be used for protein identification (Lacerda *et al*., 2007; Fig. 2). In addition to being an integrative component of system approaches (Fig. 1), metaproteomics presents some valuable advantages over other ‘omics’ technologies for functional analyses. Primarily, metagenomic data only account for the microbial potential of a system and do not provide any insights into microbial activity. On the other hand, metatranscriptomics is one step closer to the identification of active metabolic pathways but does not allow for translational regulation to be taken into consideration; indeed, a lack of correlation between mRNA levels and proteins levels has been previously documented (Gygi *et al*., 1999; Pradet-Balade *et al*., 2001). Finally, metaproteomics provides significant insights into microbial activity together with metametabolomics, which is the study of the intermediate and end-products of cellular processes. Metagenomic data typically include numerous genes of unknown function (Ram *et al*., 2005), most likely involved in novel functional systems. Metaproteomics may be useful to identify the circumstances under which these unknown functions are required and might therefore help to elucidate which systems hold the most potential for further investigation. Metaproteomics might also prove to be valuable for the identification of key microbial activities occurring in natural environments that could be exploited in a bioengineered context. For example, the investigation of the proteins expressed within the wood termite gut (Burnum *et al*., 2011) or the sheep gut (Toyoda *et al*., 2009) aimed at identifying natural microbial processes involved in the degradation of wood and cellulose. Such microbial processes could be harnessed for the production of renewable energy from wood or grass. For the purpose of this review, we discuss the advancement of metaproteomics in the context of human biology, soil, marine and freshwater environments as well as natural and bioengineered systems.

## Protein expression in the human microbiome

In the context of human biology, the analysis of microbial community function has been investigated mainly in the intestinal tract and the oral cavity. Metaproteomic approaches in this field promise to be increasingly comprehensive because of the exponential progress in the generation of relevant genomic and metagenomic datasets. The Human Microbiome Project, which aims at characterizing the microbial communities present at several sites on the human body, has generated to date 178 full genome sequences from intestinal species (Nelson *et al*., 2010), while gut metagenomic data were recently reported for 124 European individuals (Qin *et al*., 2010). The reason behind the high activity in this field of research lies in the apparent links between microbial communities and human health and disease. Applying metaproteomic approaches in such context has the potential to lead to the identification of protein markers that may be indicative of a healthy or a diseased state. In addition, the untargeted nature of metaproteomics makes it an ideal strategy to map unforeseen interactions between the human microbial communities and its host.

Despite the wealth of available genomic and metagenomic data, only a small number of metaproteomic studies have been reported to date. One of the first attempts at characterizing protein expression from the gut revealed the difficulty of such a task in the absence of relevant sequences in the databases. Klaassens *et al*. (2007) conducted a metaproteomic analysis of faecal samples from infants using two-dimensional gel electrophoresis (2-DGE) in combination with mass spectrometry analysis (matrix-assisted laser desorption/ionization time-of-flight mass spectrometry, MALDI-TOF-MS). From 100 μg of protein, the authors could obtain more than 200 protein spots on each 2D-gel. Fifty-five spots were excised and analysed by mass spectrometry, but no protein identification could be obtained. After *de novo* sequencing was performed for four spots, 11 peptide sequences were determined, of which only one showed a high-level of similarity with a known protein (bifidobacterial transladolase). More recently, the identification of 2214 proteins from human faecal samples was reported using a shotgun metaproteomic approach (Verberkmoes *et al*., 2009a). In this study, proteins were extracted from about 25 mg of microbial cells recovered from faecal material of twins, and the resulting peptide mixture was analysed using 2D-liquid chromatography-tandem mass spectrometry (2D- nano-LC-MS/MS). Mass spectra were searched against four databases, which all included two unmatched metagenome datasets (Gill *et al*., 2006), and were supplemented with relevant sequences such as *Bacteroides* spp. genomes (Verberkmoes *et al*., 2009a). One of the major breakthroughs from this study was the demonstration that unmatched metagenomes can be used to identify protein expressed from mixed microbial consortia (Verberkmoes *et al*., 2009a). This finding was quite remarkable because each individual possesses a unique gut microbial communities (Zoetendal *et al*., 2006). Another interesting result reported by Verberkmoes *et al*. (2009a) was the observation of a clear discrepancy in the distribution of clusters of orthologous groups (COG) categories between the metaproteome and the metagenome. This emphasizes the advantages of using a metaproteomic approach, whereby the measure of expression and translation of a gene product can be achieved, over a metagenomic approach, which is only indicative of the potential of a gene to be expressed and translated. More recently, an iterative workflow has been successfully developed to achieve an optimized use of the continuously growing genomic and metagenomic databases and has led to the identification of up to 5000 peptides from one sample (Rooijers *et al*., 2011). In this study, proteins were extracted from the microbial fractions recovered from faecal samples of two individuals. The extracted proteins (50 μg per sample) were separated by one-dimensional polyacrylamide gel electrophoresis (1D-PAGE) prior to LC-MS/MS analysis. Metagenomic data matched to these two faecal samples were also collected. Metagenome annotation has been identified as the main shortcoming for the utilization of such data for protein identification (Keller & Hettich, 2009; Verberkmoes *et al*., 2009b; Röling *et al*., 2010). The strength of the iterative workflow proposed by Rooijers *et al*. (2011) is the use of nonannotated, unassembled metagenome sequences in conjunction with robustly annotated genomes from single microorganism. This iterative workflow could be systematically applied to further metaproteomic analyses provided that metagenome sequences representative of the system under investigation are available.

The characterization of the microbial communities from the human oral cavity is also gaining momentum with the Human Oral Microbiome Project, which has made available an extensive 16S rRNA gene sequence database from oral microorganisms and with increasing efforts to determine the genetic diversity of oral microbial communities (Lazarevic *et al*., 2009; Dewhirst *et al*., 2010; Crielaard *et al*., 2011). The oral microbiota has been associated with infectious diseases such as periodontitis and tonsillitis, but also with systemic diseases such as stroke and pneumonia (Joshipura *et al*., 2003; Awano *et al*., 1995; Dewhirst *et al*., 2010). The microbial community of the oral cavity has also been suggested as a diagnostic marker for oral cancer, specifically targeting the abundance of three bacterial species; *Capnocytophaga gingivalis*, *Prevotella melaninogenica* and *Streptococcus mitis* (Mager *et al*., 2005). However, up until now, the characterization of the microbial communities from the human mouth has been mainly directed at determining the diversity more than the functionality of such communities. Indeed, when metaproteomic data are generated for oral cavity samples, most studies so far only reported on the resulting phylogeny obtained from peptide species assignment and did not investigate the functions of the proteins identified (Guo *et al*., 2006; Xie *et al*., 2008; Grant *et al*., 2010). Realizing the potential of the generated data, some of the authors involved in the study of Xie *et al*. (2008) revisited their results and provided a thorough analysis of the functions represented by the proteins expressed in the oral cavity (Rudney *et al*., 2010). In that respect, this study constitutes the only true metaproteomic analysis conducted on the human oral microbiota. The authors successfully developed a three-step method to effectively characterize the proteins expressed in salivary samples and identified over 1000 human proteins and 139 microbial proteins from 200 μg of protein extract (Xie *et al*., 2008; Rudney *et al*., 2010). The oral microbial mixed community was found to be metabolically active and mainly involved in the transport of carbohydrate. One of the major hurdles however in this field of research is the absence to date of oral metagenomic data. Once such data become available, future comparative metaproteomic studies should aim at characterizing community function in response to disease to identify functional biomarkers that could be used to develop diagnostic tests.

## Protein expression in soil

Soil is widely regarded as a difficult medium for the analysis of microbial community structure and function. Although soil contains an abundance of microorganisms (∼10^9^ cells g^−1^ soil; Rosselló-Mora & Amann, 2001), the high species diversity, particularly of uncultured organisms, has hindered analytical progress. In addition, the presence of inhibitory substances, such as humic acids, has proved difficult for the extraction of nucleic acids and proteins from soil, without compromising downstream processing methods, or resulting in protein degradation (Solaiman *et al*., 2007). As a result of these issues, the success of soil metaproteomics has been somewhat limited. Initial studies focused on investigating the optimization of protein extraction protocols that remove interfering compounds without inhibition of successive analysis (for details, see review from Bastida *et al*., 2009; Chen *et al*., 2009). One of the earliest reports of attempted identification of soil proteins from excised 1D-PAGE bands was carried out by Schulze *et al*. (2005). This study investigated the extracellular protein fraction from soil microparticles using LC-MS/MS analysis. The resulting mass spectra were searched against the NCBI database, which led to the identification of 75 proteins involved in the degradation of biological matter and included cellulases, collagenases, lignin phenoloxidases and laccases. Fifty per cent of the identified proteins were assigned to bacterial species, while the remaining proteins originated mainly from plants, fungi, vertebrates and nematodes. An interesting observation from this study was the redundancy of protein function as similar proteins were expressed by a range of species. For example, cellulases were expressed by three different bacteria and one fungus. More recently, a direct protein extraction method led to the identification of 716 proteins from 5 g of soil (Chourey *et al*., 2010). In this study, the protein fractions were analysed using 2D-nano-LC-MS/MS, and the databases used for protein identification consisted of all the fully sequenced microbial genomes from the Integrated Microbial Genomes database in addition to an unmatched metagenome dataset from agricultural soil (Tringe *et al*., 2005). The metaproteome was found to reflect survival strategies such as bacterial spore formation, probably to overcome the extended summer drought typical of the soil under investigation (Chourey *et al*., 2010). Another study (Wang *et al*., 2011) individually analysed the rhizospheric soil metaproteome associated with rice, sugar cane and the flowering plant *Rehmanniae*. Separation of extracted proteins by 2-DGE allowed the visualization of approximately 1000 individual proteins on each gel, with 538 common to all three samples, from which a subset of 287 spots were randomly selected for protein identification. Using MALDI-TOF/TOF followed by MS/MS and the NCBI database to search the resulting mass spectra, 189 spots were successfully identified, including 107 proteins attributed to plants, 72 to bacteria and fungi and 10 to fauna. The identified proteins were associated with a wide range of functional categories including, amongst others: energy metabolism, protein turnover, amino acid biosynthesis and proteins involved in resistance mechanisms.

In the soil environment, metaproteomics can be used to understand complex community interactions associated with *in situ* bioremediation of contaminated soil sites. Earlier studies such as Renella *et al*. (2002) and Singleton *et al*. (2003) focused on metal contaminants such as cadmium, while more recent studies moved towards organic contaminants such as 2,4-dichlorophenoxy acetic acid (Benndorf *et al*., 2007), toluene (Williams *et al*., 2010) and diesel fuel (Bastida *et al*., 2010). Benndorf *et al*. (2007) conducted metaproteomic analyses on soil microcosms to investigate the degradation of 2,4-dichlorophenoxy acetic acid (2,4-D). Soils were percolated with 2,4-D solution to promote the development of indigenous degrading species, and one of the microcosms was also augmented with a mixture of bacteria known to degrade 2,4-D. Proteins were extracted from 5 g of soil and separated by 1D-PAGE, prior to excision of protein bands. Protein identification was carried out using nano-LC-electrospray ionization (ESI) source-MS/MS, and searching the resulting mass spectra against the NCBI database. A total of only four proteins were identified, and the low level of protein identification was attributed to the difficulties encountered during soil protein extraction because of the presence of interfering humic compounds. Despite the limited number of proteins identified, the authors could conclude that the autochthonous soil community showed similar 2,4-D degradation capability as that of the augmented soil sample based on the function of the identified soil proteins (specifically, 2,4-dichlorophenoxyacetate dioxygenase), as well as the monitoring of 2,4-D degradation using HPLC separation combined with UV detection (Benndorf *et al*., 2007). More recently, changes in the metaproteome of a soil microbial community were investigated in response to glucose and toluene amendment (Williams *et al*., 2010). The microbial fraction was isolated from 5 g of soil, and the corresponding extracted proteins were separated by 1D-PAGE and analysed by MALDI-TOF/TOF followed by MS/MS. This study detected 187 proteins in total amongst which 47 were identified when searching mass spectra against the NCBI database. Proteins specifically associated with known toluene degradation pathways could not be identified, but the metaproteomic data overall reflected microbial adaptation to the presence of toluene, noted by the expression of stress-related proteins (Williams *et al*., 2010). One of the major limitations of this study, however, is the lack of measurement of the level of toluene degradation, which consequently does not allow for any conclusions regarding the toluene degrading capabilities of the soil community.

Also focusing on bioremediation, Bastida *et al*. (2010) compared the protein content of an artificially hydrocarbon-amended soil with an unpolluted control. Proteins were extracted from the microbial fraction recovered from 10 g of soil and either separated by 1D-PAGE followed by LC-ESI-MS, or by direct LC-ESI-MS. Mass spectra were searched against the bacterial entries of the NCBI database. The majority of the 42 identified proteins were related to cellular metabolism, indicating high microbial activity both in the control and the polluted soil; however, no specific known hydrocarbon degradation enzyme could be detected in the polluted soil (Bastida *et al*., 2010). Once again in this study, no measurement of the level of pollutant degradation was reported, and therefore, no information regarding the degrading capabilities of the soil communities was provided.

Overall, the field of soil metaproteomics is still in its infancy, mainly because of the technical difficulties typical of this environment. Improvements in extraction protocols are reported regularly, and even though only one soil metagenomic study has been published to date (Tringe *et al*., 2005), it has been largely underused. Strikingly, the highest number of proteins identified by soil metaproteomics so far (716 proteins) was based on the use of databases including an unmatched soil metagenome (Chourey *et al*., 2010). This study however was still a proof of concept, where the authors aimed to demonstrate the technical feasibility of soil metaproteomics and did not really discuss identified protein functions.

An interesting alternative strategy to metaproteomics, which has been employed in soil, is the use of functional metagenomics, where metagenomic DNA is expressed in a surrogate host, typically *Escherichia coli*. The resulting clones are subsequently screened for specific activities such as antibiotic resistance (Allen *et al*., 2009; Donato *et al*., 2010). Functional metagenomics has proven very useful for the identification of novel enzymes involved in antibiotic resistance and can be used as a targeted strategy to uncover specific metabolic activities. However, functional metagenomics does not provide insights into *in situ* processes, but reveals the function of metagenomic DNA in *E. coli*.

Future studies in this field should aim at combining metagenomics with metaproteomics within the soil environment, which is largely dominated by uncultured microorganisms. Taking advantage of the only soil metagenome available to date (Tringe *et al*., 2005), attempts should be made to reanalyse existing mass spectra using databases inclusive of this metagenome. Furthermore, the pending availability of soil metagenomic data as instigated by the TerraGenome Project (Vogel *et al*., 2009) should considerably improve the success of protein identification from soil environmental samples. In addition, when investigating contaminant degradation, efforts must be made to ensure that pollutants levels are monitored throughout the duration of the experiment to clarify whether the nonidentification of proteins associated with contaminant degradation is because of the lack of degradation activity or is masked by more abundant processes. Remarkably, despite the low number of identified proteins, Benndorf *et al*. (2007) could correlate the measured contaminant degradation with the presence of specific pollutant degrading proteins in their soil samples. This might imply that other bioremediation studies (Bastida *et al*., 2010; Williams *et al*., 2010), which did not include the monitoring of the level of contaminants in their experimental setup, might have been investigating pollutants at concentrations that did not allow for the soil community to utilize its degrading capabilities.

## Protein expression in marine and freshwater

Aquatic regions of the world harbour significant microbial populations with an average of ∼2.5 × 10^6^ cells ml^−l^ of seawater (Kan *et al*., 2005). These natural mixed microbial communities play crucial roles in essential processes, such as carbon and nitrogen cycling and organic matter decomposition (Maron *et al*., 2006). Metagenomic approaches have revealed the extensive microbial diversity and metabolic potential of marine and freshwater mixed communities (Venter *et al*., 2004; Rusch *et al*., 2007; Ng *et al*., 2010). These metagenomics datasets provide an ideal platform for metaproteomics, which is a growing field both in marine and freshwater ecosystems.

Metaproteomics aims at capturing the protein expression profile at the point of sampling, and thus, limiting interferences with the natural microbial consortia in samples is of vital importance. Typically, once water samples are collected, the microbial fraction is immediately concentrated prior to storage pending protein extraction. To stop further protein expression in response to sample handling conditions, strategies such as chemical fixation (Kan *et al*., 2005) or microwave fixation (Mary *et al*., 2010) have been employed. The majority of studies, however, transfer concentrated pellets directly to storage at −80 °C or employ liquid nitrogen flash freezing (Sowell *et al*., 2009, 2011; Morris *et al*., 2010).

As discussed earlier in the human biology context and in the soil environment, identification of the protein expressed by mixed microbial consortia has proven to be challenging without the availability of relevant metagenomic data. Kan *et al*. (2005) conducted the first metaproteomic analysis of a complex aquatic microbial community from Chesapeake Bay using 2-DGE in combination with both MALDI-TOF and LC-MS/MS. From 100 μg of extracted proteins, the authors could detect over 200 protein spots on each 2D-gel. Forty-eight spots were excised, from which no protein identification could be obtained using MALDI-TOF analyses. Seven of the 48 spots were further analysed using LC-MS/MS and *de novo* sequencing, which led to the identification of three proteins that shared homologies with proteins annotated from the Sargasso Sea metagenome (Venter *et al*., 2004). More recently, a metaproteomic study investigated the functionality of specific groups of microorganisms known to be abundant in the oligotrophic Sargasso Sea (Sowell *et al*., 2009). Protein extracts from microbial cells recovered from two samples of ∼230 L of surface seawater were analysed using LC-MS/MS. The resulting mass spectra were searched against three synthetic metaproteomes comprising, respectively, the SAR11 clade, *Prochlorococcus* and *Synechococcus* genomic fragments from the Sargasso Sea metagenome (Venter *et al*., 2004). This approach led to the identification of 236 SAR11 proteins, 402 *Prochlorococcus* proteins and 404 *Synechococcus* proteins, which were found to largely reflect cellular adaptations to stringent environmental conditions under which the microorganisms are competing for nutrients (Sowell *et al*., 2009). A comparative metaproteomic study investigated the membrane proteins expressed by the mixed microbial communities from surface waters in the South Atlantic (Morris *et al*., 2010). This study compared metaproteomic profiles over a large geospatial natural gradient extending from an oligotrophic gyre to a nutrient-rich coastal upwelling region. Microorganisms were recovered from 10 samples of 100–200 L each, and the enriched extracted membrane protein fractions were analysed by LC-MS/MS. The resulting mass spectra were searched against an extensive database from the Global Ocean Sampling (GOS) metagenomic project containing over 600 000 predicted proteins (Rusch *et al*., 2007; Yooseph *et al*., 2007). In total, this study identified 2273 proteins with 428 ± 158 distinct membrane proteins per sample (Morris *et al*., 2010), compared with 1042 proteins identified when whole protein extracts were analysed (Sowell *et al*., 2009). To illustrate the need for relevant metagenomic sequences when conducting metaproteomics analyses, Morris *et al*. (2010) searched their mass spectra against the GenBank database and recorded a staggering 6.2-fold decrease in peptide identification compared with searching against the extensive Global Ocean Sampling database. Overall, this study reported different metabolic activities as indicated by protein expression along the natural nutrient gradient investigated. Specifically, Ton-B transport systems, known to use a proton motive force for nutrient membrane translocation, were enriched in the nutrient-rich coastal samples, while porins and permeases were preferentially expressed in the oligotrophic open ocean samples. In addition, urea ABC transporters and photosystem proteins were more abundant in the open ocean, when compared with the coastal area. Overall, the authors concluded that the membrane metaproteomic data and derived species assignment reflected well the microbial biodiversity and physicochemical characteristics of the oligotrophic open ocean and nutrient-rich environments investigated (Morris *et al*., 2010).

Another study employed metaproteomics to investigate the functionality of the marine microbial communities in the nutrient-rich Oregon coastal seawater upwelling region (Sowell *et al*., 2011). The marine microorganisms were isolated from ∼100 L of surface seawater, and the extracted whole protein fractions were analysed by 2D-nano-LC-MS/MS. The generated mass spectra were searched against a database composed of the predicted proteins from the GOS project (Rusch *et al*., 2007) that was edited to contain only the sequences found in similar natural environments, as well as additional genome sequences from two Oregon coastal isolates (Sowell *et al*., 2011). Overall, this study identified 481 unique protein families, with 29% of the total detected spectra related to transport functions. The types of transport proteins identified in this nutrient-rich environment (e.g. specific for nitrogen, carbon and sulphur containing compounds) differed from those detected in the oligotrophic Sargasso Sea (e.g. specific for phosphate and phosphonate, known to be abundant during phosphorus starvation; Sowell *et al*., 2009). Differential protein expression as a response to natural nutrient gradients within the marine environment was also observed in the South Atlantic ocean (Morris *et al*., 2010).

In freshwater environments, two recent studies investigating microbial community functions in Ace Lake Antarctica directly combined metagenomics with metaproteomics (Ng *et al*., 2010; Lauro *et al*., 2011). Ng *et al*. (2010) targeted a dominant green sulphur bacterium (*Chlorobioaceae*) known to be prevalent at specific depths (12–14 m) in the water column, while Lauro *et al*. (2011) investigated the microbial communities at work throughout the water column (5–23 m). In both studies, microbial fractions were recovered from 1- and 10-L samples obtained after drilling the ice cover of the lake. Whole protein extracts were analysed using 1D-PAGE followed by LC-MS/MS. Mass spectra were directly searched against the assembled metagenome from the same sample (Ng *et al*., 2010; Lauro *et al*., 2011).

Metagenomic analysis of samples from 12.7 m depth of Ace Lake assigned 76% of the predicted open reading frames (ORF) to a single species of green sulphur bacterium, (*C*-Ace; Ng *et al*., 2010), highlighting the predominance of this organism in this environment. The resulting composite genome was then directly used to construct a database for searching metaproteomic mass spectra to inform on the functional activities of *C*-Ace at the time of sampling. This approach led to the identification of 504 proteins corresponding to ∼31% of the total predicted proteome of *C*-Ace (Ng *et al*., 2010). As observed in the context of the gut microbiome (Verberkmoes *et al*., 2009a), the comparison of the distribution of COG categories between the metaproteome and the metagenome revealed that some functional categories were misrepresented in the metaproteome. Specifically, proteins involved in translation and energy production and conversion were statistically overrepresented, while amongst others, proteins involved in defence mechanisms and inorganic ion transport and metabolism were underrepresented. In addition, the analysis of genes and corresponding proteins that did not have any orthologues in known green sulphur bacteria gave some insights about potential adaptive mechanisms in *C*-Ace. Evidence of a requirement for specific polysaccharides structures was attributed to cold adaptation, while the presence of genes encoding DNA restriction and modification system could present a protective advantage against bacteriophages. Also worth noting was the construction of a pathway for sulphide oxidation for *C*-Ace, and the lack of evidence for assimilatory sulphate reduction suggesting the strict dependence of *C*-Ace on sulphate-reducing bacteria, located at 14 m depth in Ace Lake (Ng *et al*., 2010). To conclude, the authors could access the proteins necessary for the success of *C*-Ace in Ace Lake under cold, oligotrophic, oxygen limited and extreme light conditions and therefore access the biology of this organism in its natural habitat (Ng *et al*., 2010). The structure and functions of the microbial communities in Ace Lake were further investigated throughout the water column by sampling from six depths with the aim of capturing the interactions between microbial populations that defined nutrient cycling (Lauro *et al*., 2011). The metagenomic analysis could assign ∼28% of open reading frames to COG categories, while the metaproteomic study identified 1824 proteins. Interestingly, phylogenetic diversity was found to increase with depth and was associated with an increased amount of hypothetical proteins, which accounted for 67% of the identified proteins at 23 m depth. In addition, most of these proteins did not match any orthologues from known microorganisms and could belong to novel functional pathways (Lauro *et al*., 2011). Combining the physicochemical data available for Ace Lake with matching metagenomic and metaproteomic data, Lauro *et al*. (2011) could describe the carbon, nitrogen and sulphur cycles throughout the water column. Briefly, for the carbon cycling, cyanobacteria were found to carry out aerobic carbon fixation in the upper stratum of the lake, anaerobic carbon fixation occurred further down the water column and was performed by green sulphur bacteria conjointly with sulphate-reducing bacteria, while fermentation processes were thought to take place at the bottom of the lake. In addition, remineralization of particulate organic carbon to dissolved organic carbon was found to occur at the surface of the lake with further heterotrophic conversion carried out by *Actinobacteria* and members of SAR11 clade. In the lower stratum of the lake, remineralization of particulate organic carbon was thought to result from the joint activities of fermentative, sulphate-reducing and methanogenic microorganisms ultimately leading to the production of CO_2_ and CH_4_. Carbon monoxide oxidation was also thought to be an important energy generation pathway throughout the water column as indicated by the detection of CO dehydrogenase genes (Lauro *et al*., 2011). The nitrogen cycle was found to involve nitrogen assimilation throughout the lake, as indicated by the detection of glutamine and glutamate synthetases in the metaproteome, with remineralization localized at the lower stratum. Interestingly, no evidence of nitrification occurring throughout the water column could be detected. Indeed, the metagenome did not include any ammonia oxidation genes or any signatures of known nitrifying bacteria or archaea. As Ace Lake is associated with continuous low level of nitrate, the authors suggested that the absence of nitrification might be a strategy to conserve bioavailable nitrogen (Lauro *et al*., 2011). Finally for the sulphur cycle, green sulphur bacteria were found to consume the sulphide produced by the sulphate-reducing bacteria, to generate sulphate and thus providing the replenishment of the sulphate-reducing bacteria substrate and ensuring the continuous turnover of sulphur compounds in the lake. Even though genes for assimilatory sulphate reduction were detected in the metagenome, no evidence of their expression could be found in the metaproteome. However, dissimilatory sulphide reduction was found to be an active pathway as indicated by the identification of proteins from sulphide reductase complex from green sulphur bacteria (Lauro *et al*., 2011). Because of the crucial role seemingly played by *C*-Ace in the lake, as outlined by metagenomics and metaproteomics, and the low number of viral particles where this bacterium is located (12.7 m depth), the authors suspected that *C*-Ace was not following the previously developed model of viral/population dynamics in aquatic environments (Rodriguez-Brito *et al*., 2010). The authors attributed this unusual observation to the influence of extreme light conditions on the microorganism biology. To test their hypothesis, a mathematical model was developed, where the growth of *C*-Ace was intrinsically linked to the light intensity in the lake (Lauro *et al*., 2011). From this, the authors could predict that the persistence of *C*-Ace in the lake was because of the absence of phage. By incorporating this prediction with the information deduced from metagenomics and metaproteomics, it was concluded that the emergence of phage predators could deplete the Ace Lake of green sulphur bacterial populations, which, because of their central role in the recycling of carbon, nitrogen and sulphur, would have severe consequences for the whole lake community (Lauro *et al*., 2011). Overall, these two studies (Ng *et al*., 2010; Lauro *et al*., 2011) focused on the interpretation of the data generated using metagenomics and metaproteomics and are quite unique in that aspect as they did not stop at the demonstration of the feasibility of applying such technologies to Ace Lake but fully used their results to gain insights into the biology of this ecosystem.

In most environments, the difficulties associated with attempting to characterize the metaproteome without matched or unmatched metagenome sequences have been demonstrated. However, this does not apply when investigating natural environments largely dominated by one species, where protein identification can be obtained using a single sequenced relevant microbial isolate (Habicht *et al*., 2011). Conversely, one could argue that these types of study do not analyse the metaproteome but simply investigate the microbial behaviour of one species within its natural environment. Such a study identified 1321 proteins from *Chlorobium clathratiforme* (species representing more than 50% of the bacterial population) throughout the water column in Lake Cadagno, Switzerland (Habicht *et al*., 2011). Interestingly, when searching the mass spectra generated by LC-MS/MS against all *Chlorobi* genomes, an additional 350 proteins could be identified, revealing the presence of species not previously detected in the lake. A search against the entire UniProt database led to the identification of a further 120 proteins from various species. Such a study demonstrates that, if the microbial communities investigated are largely dominated by known species, LC-MS/MS analysis can lead to extensive results in the absence of relevant metagenomic data. However, even if such results are valid, they are nonetheless biased towards dominant species and might not be suitable for integration in system approaches (Fig. 1).

## Protein expression in natural and bioengineered systems

Scientists have long known the significance of the role played in the environment by mixed microbial communities. However, the exact processes carried out by these natural consortia are far from resolved. Even in relatively simple natural biofilms, the expression of thousands of proteins is required to enable survival and growth, including proteins involved in nutrient metabolism, stress response and environmental signalling (Ram *et al*., 2005). One such biofilm community, originating from an acid mine drainage system within the Richmond mine at Iron Mountain, California, has been extensively studied (Ram *et al*., 2005; Lo *et al*., 2007; Belnap *et al*., 2010; Denef *et al*., 2010; Mueller *et al*., 2010, 2011; Wilmes *et al*., 2010; Pan *et al*., 2011). Indeed, one of the first publications investigating mixed microbial consortia from this site, Ram *et al*. (2005), is widely considered as a breakthrough study in environmental metaproteomics. Even though very significant, most of these studies however (Lo *et al*., 2007; Denef *et al*., 2010; Wilmes *et al*., 2010; Mueller *et al*., 2011) do not really fall under the umbrella of metaproteomics, but as in the case of Habicht *et al*. (2011), they investigate the physiology of one dominant species within its natural habitat. Nonetheless, these approaches collectively participated in important methodological advances and will be briefly discussed here.

Lo *et al*. (2007) demonstrated the use of proteomics to determine genomic divergences between uncultivated microorganisms and closely related sequenced species within the acid mine drainage biofilm. Denef *et al*. (2010) employed this strategy to differentiate between two closely related genotypic groups of *Leptospirillum* group II (99.7% similarity between 16S rRNA gene sequences). While one genotypic group typically prevails in early-stage biofilm, the other is dominant in the later stages of biofilm formation. To investigate this ecological divergence, proteomic data previously published from 27 samples (Denef *et al*., 2009) were analysed for the determination of ecologically preferential functions. The authors identified a subset of proteins that could be associated with the observed ecological divergence. Specifically, proteins originating from an early-stage biofilm were found to be mainly involved in co-factor biosynthesis and motility, while proteins associated with energy production and conversion emerged as biofilm maturity progressed (Denef *et al*., 2010). Applying a similar strategy, Wilmes *et al*. (2010) focused on the genomic divergence of two dominant species within the acid mine drainage biofilm, namely *Leptospirillum* groups II and III, using a combination of proteomics and metabolomics. A total of 765 proteins and 3740 metabolites were detected across 14 biofilm samples. Each of the two *Leptospirillum* groups investigated could be associated with a distinct cluster of proteins and metabolites, indicating genomic divergence between the two bacteria. From their data, the authors could identify a limited niche overlap and a low level of interspecies competition, suggesting separate resource utilization for each species within the biofilm (Wilmes *et al*., 2010).

Investigating further the acid mine drainage biofilm, Mueller *et al*. (2010) combined metaproteomics with geochemical and biological data to investigate the behaviour of the biofilm microbial communities along environmental gradients. The analysis of 28 biofilm samples led to the identification of a total of 6296 proteins and differences in protein expression was found to correlate with environmental parameters (Mueller *et al*., 2010). For example, temperature at the time of sampling was found to impact on levels of cold-shock proteins and proteins involved in fatty acid biosynthesis, expressed at a lower level and at a higher level with increasing temperature, respectively. In addition, a shift in the metaproteome was observed from ribosome biosynthesis and transcription in the early stage of biofilm formation to environmental signalling, chemotaxis and biosynthesis of extracellular components in the mature biofilm (Mueller *et al*., 2010). In a subsequent study, Mueller *et al*. (2011) focused their investigations on the initial dominant species, *Leptospirillum* Group II, and its evolving protein expression throughout the development of the biofilm community. Using replicate samples and analysing membrane and cytoplasmic protein fractions, this study aimed at identifying key proteins that could be used as biomarkers to estimate biofilm maturity (Mueller *et al*., 2011). The authors identified a total of 4107 distinct proteins, corresponding to ∼45% of the predicted proteome of *Leptospirillum* Group II. Changing protein abundance patterns were successfully determined as a function of biofilm maturity. For example, the metabolism of simple carbon compounds was found to dominate early growth stage biofilms, while the metabolism of complex carbohydrates increased in late stages of biofilm formation (Mueller *et al*., 2011). Although Mueller *et al*. (2011) briefly mentioned the use of ^15^N for quantification of expressed protein, an earlier study by Belnap *et al*. (2010) investigated quantitative proteomic analysis more thoroughly to compare a laboratory-grown biofilm with a natural biofilm. The aim of this study was to validate the use of laboratory model systems for the investigation of ecological hypotheses. In total, the relative quantification of over 2500 proteins between natural- and laboratory-grown biofilms was determined. This comparison showed that improvements in laboratory culturing conditions resulted in the decrease of metabolic stress protein expression, such as proteins involved in defence mechanisms and oxidative damage repair, and this correlated with increased community growth rates (Belnap *et al*., 2010). This study highlighted the relevance of using model systems under laboratory controlled conditions to gain some understanding of natural microbial processes. A subsequent study by Pan *et al*. (2011) used this laboratory culture model to successfully develop a high-throughput stable isotope probing (SIP) method. Employing this new technique, the authors could trace the incorporation of ^15^N into thousands of proteins expressed by a mixed microbial community. Also applying SIP technology, Bastida *et al*. (2011) quantitatively traced the movement of ^13^C-benzene between trophic levels, specifically from bacteria to eukaryotic organisms, such as *Chironomus* sp. larvae. SIP-proteomics methods could prove very valuable to track down fluxes of ^13^C or ^15^N in mixed microbial communities. However, such setups could not be applied *in situ* and are limited to laboratory-based models including microcosms.

Although the incorporation of labelled carbon and nitrogen has been investigated in the context of laboratory-grown mixed anoxic communities (Jehmlich *et al*., 2008), to the best of our knowledge, no protein-based SIP analysis of complex, engineered systems has been yet undertaken, which could allow for optimization of economically significant processes. However, metaproteomic studies of engineered processes, such as activated sludge systems, are extensive (Wilmes & Bond, 2006; Park & Novak, 2007; Park *et al*., 2008a, b; Abram *et al*., 2011). Activated sludge systems are principally employed for the treatment of wastewater in developed countries. The aggregation of microbial biomass to form well-settling flocs has been identified as a key parameter for the successful operation of wastewater treatment plants. The study of the metaproteome of laboratory-scale-activated sludge systems has been divided into two approaches: analysis of the extracellular polymeric substances (EPS) that bind the microbial cells together, and analysis of the protein fraction located within the microbial cells themselves.

Researchers from the University of Massachussetts/Virginia Tech focused on the extraction and analysis of the protein component of EPS. Their first study (Park & Novak, 2007) identified that EPS bound to different cations require different extraction methods, which resulted in the extraction of ‘sub-proteome’ components of the activated sludge sample. Focusing on the requirement for the reduction of volatile solids to 38% prior to land spreading of digested sludge (US EPA, 2003), Park *et al*. (2008a) demonstrated the selective degradation of divalent cation-bound proteins and aluminium-bound proteins by aerobic digestion, but not by the anaerobic treatment process, while the opposite was true of organic matter bound with ferric iron. This is of significant industrial importance, as it specifically indicates by protein analysis, that a two-step treatment process, featuring both anaerobic and aerobic stages, may be required for enhanced sludge treatment.

Next, Park *et al*. (2008b) attempted to identify proteins that could serve as biomarkers to monitor facility operations in activated sludge processes. Eleven intense bands from 1D-PAGE were investigated using LC-MS/MS and the whole NCBI database. Seven of these bands contained identifiable proteins associated with bacterial defence, cell appendages, cell surface outer membrane proteins, intracellular materials and influent sewage proteins. Interestingly, of the bands that did not return any positive matches, identifiable amino acid fragmentation patterns were obtained, indicating that these proteins are likely to originate from unsequenced microorganisms in full-scale-activated sludges, again indicating the requirement for metagenomic sequence data. As seen in other environments (human microbiome, soil, marine and freshwater), the use of unmatched metagenome sequences may have improved the success of protein identification in the above investigation. A short follow-up study analysed one of the samples from Park *et al*. (2008b) over the course of a 30-day batch digestion (Park & Helm, 2008). Three 1D-PAGE bands, additional to those studied in Park *et al*. (2008b), were investigated by LC-MS/MS using the whole NCBI database. All three bands (1) were associated only with divalent cations, (2) emerged during the batch anaerobic digestion of the activated sludge and (3) were identified as different subunits of methyl-coenzyme M reductase, a key enzyme for methane formation in methanogenic archaea, and in this case associated with *Methanosarcina barkeri*.

The intracellular microbial protein fraction of activated sludge has been investigated by a separate group of researchers. Wilmes & Bond (2006) applied metaproteomics to activated sludge originating from the end of the anaerobic and aerobic cycles of the same sequencing batch reactor (SBR) during stages of enhanced biological phosphorus removal (EBPR) and non-EBPR (nEBPR). From 100-mL sludge samples, separation by 2-DGE allowed the detection of an average of 665 spots per gel, although spot patterns differed significantly between EBPR and nEBPR samples. Furthermore, proteins patterns detected from the EBPR sludge samples were more consistent between aerobic and anaerobic stages, indicating the development of a more stable microbial community, which was not noted in the nEBPR samples. Although no attempt at protein identification was undertaken, the importance of this study must not be underestimated, as the demonstration of a statistically different protein expression between a functioning and a nonfunctioning system justifies the investigation of the metaproteome for identification of performance issues associated with full-scale-activated sludge processes.

A subsequent study by this group focused on the identification of proteins separated by 2-DGE. Using a combination of MALDI-TOF/MS and quadrupole-TOF MS/MS, and searching against three unmatched EBPR metagenomic databases, Wilmes *et al*. (2008a) identified 38 of 111 excised protein spots, corresponding to 33 unique proteins. It is suggested that the low identification rate (41%) may be due to strain variation between the sample sludge (from a UK SBR) and the three metagenomic databases employed (two from the United States and one from Australia). Possibly recognizing the technical limitations of 2-DGE, Wilmes *et al*. (2008b) then carried out analysis of extracted proteins directly via 2D nano-LC, followed by MS/MS analysis. Peptide tandem mass spectra were matched *in silico* to the three separate sludge metagenomic databases employed in Wilmes *et al*. (2008a), and against a combined database of the three metagenomic datasets. This extensive analysis resulted in the identification of a total of 5029 proteins, with 36% associated with *Accumulibacter phosphatis*, a major polyphosphate accumulating organism.

While activated sludge technology is commonly employed in the field of sewage treatment, anaerobic digestion technology is becoming increasingly popular for the treatment of recalcitrant chemical-containing wastewater streams. Jehmlich *et al*. (2010) were one of the first reported studies to investigate the metaproteome of an anaerobic community, originating from a batch sulphate-reducing enrichment culture, exposed to toluene. This study analysed 150 μg of protein by 2-DGE and identified 202 proteins from 236 excised gel spots using nano-LC-ESI-MS/MS analysis and searching the resulting mass spectra against the bacterial entries of the NCBI database. Identified proteins were found to be associated with both sulphate reduction and toluene degradation. Recently, the first metaproteomic investigation of granular biomass originating from a continuous anaerobic bioreactor was described (Abram *et al*., 2011). The extracted proteins were separated by 2-DGE, which resulted in the detection of 388 reproducible protein spots, of which 70 were excised. Thirty-three proteins were positively identified using nano-LC-ESI-MS/MS and searching the resulting mass spectra against both the whole NCBI database and the Trembl database. Proteins associated with the production of methane and the degradation of glucose were identified. One of the major hurdles in the field of anaerobic community function is the lack of metagenomic data, which would facilitate the identification of proteins.

## Conclusions

Overall, the field of metaproteomics is gaining momentum at an exponential rate within very diverse environments. An overview of selected studies from the ecosystems discussed in this review is shown in Table 1. Advances in metaproteomics finally allow for the consideration of the integration of such data in system approaches (Fig. 1). This was partly achieved in the aquatic environment where Lauro *et al*. (2011) combined metagenomic, metaproteomic and physicochemical data to describe the interaction between the microbial populations defining the biogeochemical cycles throughout a water column. Such an approach could feasibly be transferred to other environmental ecosystems. To date, the application of complex system approaches is still scarce and requires a coordinated experimental design that brings together expertise from each of the many technologies involved.

**Table 1:** Overview of selected metaproteomics studies

Environment	Number of peptides/proteins identified	Method	Databases	References
Human gut	NA/2214 proteins	LC-MS/MS	2 unmatched human gut metagenomes, several genomes from gut inhabitants and several nonhuman gut genome	Verberkmoes *et al*. (2009a)
Human gut	5010 peptides/NA	1D-PAGE, LC-MS/MS	Synthetic human gut metagenome (216 genomes from gut inhabitant) and 124 human gut unassembled nonannotated metagenomes	Rooijers *et al*. (2011)
Soil	NA/716 proteins	LC-MS/MS	Unmatched soil metagenome supplemented with 1606 genomes	Chourey *et al*. (2010)
Soil	NA/122	2D-PAGE, MALDI TOF/TOF MS/MS	Complete NCBInr, bacterial entries NCBInr and fungal entries NCBInr	Wang *et al*. (2011)
Marine	6533 peptides/1042 proteins	LC-MS/MS	SAR11 clade and specific microorganisms from Sargasso Sea metagenome as well as genomes from sequenced isolates	Sowell *et al*. (2009)
Marine	5389 peptides/2273 proteins	LC-MS/MS	Global Ocean Sampling combined metagenomes	Morris *et al*. (2010)
Freshwater	NA/1824 proteins	1D-PAGE, LC-MS/MS	Matched metagenomes	Lauro *et al*. (2011)
Acid mine drainage biofilm	NA/4107 proteins	LC-MS/MS	Biofilm_AMD_CoreDB database	Mueller *et al*. (2011)
Activated sludge	NA/5029 proteins	LC-MS/MS	Three distinct unmatched activated sludge metagenomes	Wilmes *et al*. (2008b)
Anaerobic digestion	NA/202 proteins	2D-PAGE, LC-ESI-MS/MS	Bacterial entries of the NCBI nonredundant database	Jehmlich *et al*. (2010)

1/2D-PAGE, one/two-dimensional polyacrylamide gel electrophoresis; LC-MS/MS, liquid chromatography-tandem mass spectrometry; MALDI-TOF, matrix-assisted laser desorption ionization-time of flight; ESI, electrospray ionization.

The technical limitations encountered throughout the metaproteomic workflow (Fig. 2) have, for the most part, been addressed in the ecosystems discussed in this review. However, it should be kept in mind that an exhaustive investigation of the entire metaproteome is unlikely due to the unfeasibility of developing a universal protein analysis protocol. Furthermore, it must be considered that a metaproteome may include intracellular, extracellular and membrane-bound proteins, and ideally, the three protein fractions should be analysed for each sample. When possible, opting for gel-free protein fractionation seems to lead to a higher level of protein identification when compared with gel-based methods. For example, when analysing the metaproteome of activated sludge, the use of 2-DGE resulted in the identification of 38 proteins (Wilmes *et al*., 2008a), while the 2D-nano-LC method led to the identification of 5029 proteins (Wilmes *et al*., 2008b). In addition, it is now apparent that metaproteomic approaches benefit from the availability of relevant metagenomic data, either matched or unmatched. As a result of this combined protocol, a new difficulty is encountered regarding the analysis and interpretation of the vast quantity of data generated. A major hurdle in the utilization of metagenomic data, impacting directly on metaproteomics, has been recognized as the assembly and the annotation of the collected genomic fragments. Rooijers *et al*. (2011) proposed an iterative workflow using nonannotated, unassembled metagenome sequences, which could be systematically used in metaproteomic investigations whenever relevant metagenomic data are available.

Future metaproteomic studies should aim to progress from proof of concept approaches to experimental designs leading to practical applications. For example, metaproteomic comparisons between healthy and diseased states within the human microbiome have yet to be carried out. Additionally, comparative *in situ* bioremediation investigations will need to be conducted to access the functional response of the natural mixed microbial communities to common pollutants. Furthermore, when investigating bioremediation processes, pollutants should be systematically monitored throughout the trials, to clarify the contaminant degradation status as a function of time. This in turn should allow for the proper assessment of the degrading abilities of the microbial communities investigated.

To conclude, the feasibility of metaproteomic studies has been successfully demonstrated in very diverse natural and engineered environments. However, only few studies to date employed this strategy to answer specific biological questions such as how complex communities define the biology of a given ecosystem (Ng *et al*., 2010; Wilmes *et al*., 2008b; Denef *et al*., 2010; Mueller *et al*., 2010; Lauro *et al*., 2011), and it is now critical to move metaproteomics forward in order for this technology to achieve its full potential. To this end, future studies must be designed with an aim towards gaining some understanding of ecological concepts, and the data generated must be adequately analysed. In addition, attempts should be made to integrate such data in the context of system approaches to allow for the prediction of functional responses to environmental stimuli.
